# Dose-Dependent Alterations of the Human Gut Microbiome During Oral Iron Supplementation: A Randomized Study in Iron-Deficient Non-Anaemic Women

**DOI:** 10.3390/nu18091399

**Published:** 2026-04-29

**Authors:** Morton G. Schubert, Anaëlle Dentand, Maximilian Karczewski, Yasser Morsy, Felix Beuschlein, Michael Scharl, Pierre-Alexandre Krayenbuehl

**Affiliations:** 1 Department of Endocrinology, Diabetology and Clinical Nutrition, University Hospital Zurich (USZ), University of Zurich (UZH), 8091 Zurich, Switzerlandfelix.beuschlein@usz.ch (F.B.); pierrea.krayenbuehl@usz.ch (P.-A.K.); 2 Department of Neurosurgery, University Hospital Zurich (USZ), University of Zurich (UZH), 8091 Zurich, Switzerland; 3 Department of Gastroenterology and Hepatology, University Hospital Zurich (USZ), University of Zurich (UZH), 8091 Zurich, Switzerland; yasser.morsy@usz.ch (Y.M.); michael.scharl@usz.ch (M.S.); 4 Department of Medicine IV, University Hospital, Ludwig Maximilian University of Munich, 80336 Munich, Germany; 5 The LOOP Zurich-Medical Research Center, 8044 Zurich, Switzerland; 6 General Practice Brauereistrasse, 8610 Uster, Switzerland

**Keywords:** oral iron supplementation, iron deficiency, gut microbiome, dose-dependent effects, gastrointestinal side effects, 16S rRNA sequencing

## Abstract

**Background/Objectives**: Oral iron supplementation is widely used to treat iron deficiency but frequently causes gastro-intestinal side effects that limit treatment adherence. Unabsorbed luminal iron has been proposed to influence intestinal microbial communities, yet the effects of different oral iron doses on the human gut microbiome remain insufficiently characterized. **Methods**: In this randomized open-label study, 30 healthy premenopausal women with iron deficiency without anaemia received either low-dose oral iron supplementation (6 mg twice daily) administered under fasting conditions or standard-dose iron supplementation (100 mg once daily) taken with a meal for four weeks. Stool samples were collected before and after treatment and analyzed using 16S rRNA sequencing to evaluate microbiome composition. **Results**: Baseline characteristics, including age, body mass index, hemoglobin concentration and serum ferritin, were comparable between groups. After four weeks of treatment, distinct alterations in gut microbiome composition were observed between the low-dose and standard-dose groups. The genera *Colidextribacter* and *GCA-900066575* decreased in the low-dose group but increased in the standard-dose group, whereas *Oscillospira* showed the opposite pattern. Gastrointestinal adverse events were reported by 87% of participants receiving standard-dose iron supplementation compared with 7% receiving low-dose iron supplementation (*p* < 0.0001). **Conclusions**: Oral iron supplementation induces dose-dependent changes in the intestinal microbiome and higher doses are associated with substantially increased gastrointestinal intolerance. These findings suggest that lower iron doses may reduce microbiome disruption and improve treatment tolerability.

## 1. Introduction

Iron deficiency is the most common cause of anaemia worldwide and affects approximately one billion people [1]. Iron deficiency without anaemia is likely even more prevalent and can lead to fatigue, cognitive impairment and reduced physical performance [2,3,4]. Iron supplementation has been shown to improve these symptoms and restore iron stores [2,5].

Oral iron supplementation, most commonly administered as conventional iron salts, remains the first-line therapy due to its low cost and established efficacy but frequently causes gastrointestinal side effects such as diarrhea, constipation and nausea [6]. These adverse effects are thought to arise partly from unabsorbed iron in the intestinal lumen. High concentrations of luminal iron can promote oxidative stress, local toxicity and potential disruption of the intestinal microbiome [7].

Intestinal absorption of non-heme iron occurs predominantly in the duodenum and proximal jejunum and is mediated by reduction of ferric to ferrous iron, uptake via divalent metal transporter 1, and basolateral export through ferroportin. This process is tightly regulated by the hepatic hormone hepcidin, which induces ferroportin internalization and thereby reduces intestinal iron transfer into the circulation. At higher oral iron doses, fractional absorption is relatively low and dose-dependent hepcidin induction may further reduce iron uptake, increasing the amount of unabsorbed luminal iron available to interact with the intestinal microbiota. This provides a biologically plausible mechanism by which oral iron dose may influence both gastrointestinal tolerability and gut microbial composition [8].

Iron availability is a key determinant of microbial growth within the intestinal ecosystem. Many bacterial species require iron acquisition mechanisms to proliferate and increased luminal iron availability may therefore alter microbial competition and community composition [9,10]. With increasing recognition of the gut microbiome’s role in human health, the potential effects of commonly prescribed medications on microbial composition have become an important area of investigation.

Current clinical guidelines recommend oral iron doses of approximately 50–100 mg elemental iron per day for the treatment of iron deficiency [11]. However, intestinal absorption at these doses is relatively low, with approximately 10% of administered iron being absorbed [12]. As a result, substantial quantities of iron remain within the intestinal lumen, where they may influence microbial ecology. Recent studies have also demonstrated that oral iron intake induces a dose-dependent increase in plasma hepcidin levels, which subsequently reduces iron absorption [13]. Lower oral iron doses may therefore result in more efficient absorption and reduced luminal iron exposure. Consistent with this concept, previous work from our group demonstrated that low-dose oral iron supplementation effectively improves iron stores in women with iron deficiency without anaemia while causing minimal gastrointestinal side effects [14].

Despite these findings, the influence of different oral iron doses on the human gut microbiome has not yet been systematically studied. The aim of the present study was therefore to investigate the dose-dependent effects of low-dose versus standard-dose oral iron supplementation on intestinal microbiome composition and gastrointestinal tolerability in iron-deficient non-anaemic women.

## 2. Materials and Methods

### 2.1. Study Design

This study was a prospective, randomized, open-label, single-center interventional parallel-group trial conducted at the University Hospital Zurich, Department of Endocrinology, Diabetology and Clinical Nutrition. Participants were randomly assigned to receive either low-dose oral iron supplementation (6 mg twice daily; total 12 mg/day) or standard-dose iron supplementation (100 mg once daily) for a period of four weeks. Randomization was performed using a simple allocation procedure in which participants drew one of 30 pre-prepared tickets (15 per group) at the baseline visit. A baseline visit was performed before the intervention and a follow-up visit after completion of the four-week treatment period. Stool samples were collected before and after the intervention and analyzed using 16S rRNA sequencing to assess intestinal microbiome composition. Participants were instructed to maintain their usual dietary habits and daily routines throughout the study period.

### 2.2. Outcomes

The primary endpoint of the study was the between-group difference in the change of intestinal microbiome diversity from baseline to four weeks, assessed by 16S rRNA sequencing (e.g., Shannon index). Secondary endpoints included differences in gastrointestinal adverse events between treatment groups and exploratory analyses of microbiome composition at the taxonomic level.

### 2.3. Participants

Healthy premenopausal women with iron deficiency without anaemia were recruited. Iron deficiency was defined as serum ferritin ≤30 µg/L with hemoglobin ≥117 g/L. Inclusion criteria were age >18 years, regular menstrual cycles of 23–35 days, body mass index 18–25 kg/m^2^, and *C*-reactive protein <5 mg/L. Exclusion criteria included inflammatory diseases, pregnancy, hypermenorrhea, psychiatric disorders, hypersensitivity to iron supplements, potential drug interactions, and use of dietary supplements or medications affecting iron absorption, gastrointestinal physiology, or gut microbiota composition within four weeks prior to study initiation or during the study period, including proton pump inhibitors, H2 receptor antagonists, antacids, mineral supplements, antibiotics, probiotics, laxatives, and iron-chelating agents such as tetracyclines or fluoroquinolones.

### 2.4. Study Procedures

Thirty participants (out of 89 screened) were enrolled and allocated to two treatment groups. At the baseline visit, participants were instructed on proper stool sample collection procedures. Stool samples were collected immediately before initiation of the intervention and again at the end of the four-week treatment period. Stool samples were self-collected at home using a standardized stool collection kit (OMNIgene•GUT, DNA Genotek, Ottawa, ON, Canada), which includes a collection spatula and a tube containing a stabilization buffer for microbial DNA preservation. Participants transferred a small amount of stool into the tube without opening the main container, and samples were homogenized with the stabilization solution by vigorous shaking according to the manufacturer’s instructions. The stabilization buffer allows preservation of microbial DNA at ambient temperature during transport. Samples were returned to the study center by mail using appropriate packaging. Participants received instructions on iron tablet administration and regular text messages were sent during the study to monitor treatment adherence. At the follow-up visit, adverse events were recorded and remaining iron supplements were counted to assess compliance.

### 2.5. Study Medication

Low-dose iron supplements were provided free of charge by Dr. Krayenbuehl GmbH (Pfaffensteinstrasse 32, 8118 Pfaffhausen, Switzerland) and contained 6 mg elemental iron per tablet (equivalent to 18.6 mg ferrous sulfate). Standard-dose iron supplements were obtained from Vifor AG (Rechenstrasse 37, 9001 St. Gallen, Switzerland) and contained 100 mg elemental iron per tablet. Participants in the low-dose group were instructed to take iron on an empty stomach in the morning and evening, either one hour before or two hours after meals, whereas participants in the standard-dose group were instructed to take iron once daily together with a meal.

### 2.6. Laboratory Analysis

Blood parameters including hemoglobin, iron, ferritin, transferrin and *C*-reactive protein were analyzed by the Clinical Chemistry Institute of the University Hospital Zurich. Stool samples were analyzed by the Department of Gastroenterology and Hepatology at the University Hospital Zurich in collaboration with Microsynth AG (Balgach, Switzerland).

### 2.7. Microbiome Analysis

Microbiome analysis was performed using 16S rRNA gene sequencing. Sequence data were processed using the QIIME2 pipeline (version 2023.7) for quality control, feature table construction, and taxonomic assignment using the SILVA 138 99% Naïve Bayes classifier. Alpha diversity was assessed using the Shannon index. Between-group differences were evaluated using the Mann–Whitney U test, while within-group changes from baseline to week 4 were assessed using the Wilcoxon signed-rank test. Beta diversity was assessed using weighted UniFrac distance as the primary metric; analyses using weighted Jaccard distance yielded comparable results. Differences in microbiome composition were evaluated using permutational multivariate analysis of variance (PERMANOVA) with 999 permutations. The model included treatment group and timepoint as fixed effects, with subject ID used as a strata term to account for repeated measures. Homogeneity of dispersion was assessed using PERMDISP. Differential abundance analyses across taxonomic levels (domain to species) were analyzed using the Wilcoxon rank-sum test on relative abundance data. No taxa filtering was applied. To account for multiple testing, *p*-values were adjusted using the Benjamini–Hochberg false discovery rate (FDR) method, and adjusted *p*-values < 0.05 were considered statistically significant. Rarefaction was performed to a depth of 33,197 reads per sample, corresponding to the minimum sequencing depth observed, ensuring inclusion of all samples in diversity analyses. All statistical analyses were conducted in R (version 4.3.0) and QIIME2 at a two-sided significance level of 0.05.

### 2.8. Adverse Events

Adverse events were reported by participants at the follow-up visit after completion of the four-week intervention period. Adverse events were assessed through structured interviews, during which participants were specifically asked about gastrointestinal symptoms such as nausea, abdominal pain and constipation experienced during the intervention period.

## 3. Results

### 3.1. Participant Characteristics

Thirty participants were included in the final analysis, with 15 participants assigned to each treatment group. Baseline characteristics were comparable between groups. The mean age was 23 ± 3 years in the low-dose group and 22 ± 2 years in the standard-dose group. Body mass index values were similar between groups. Hemoglobin concentrations were 136 ± 8 g/L in the low-dose group and 138 ± 7 g/L in the standard-dose group, while serum ferritin levels were 18 ± 7 µg/L and 18 ± 5 µg/L, respectively (Table 1). All participants had normal *C*-reactive protein levels at baseline.

### 3.2. Microbiome Diversity

Alpha diversity, assessed using the Shannon index, did not show significant differences between the treatment groups after the four-week intervention period.

### 3.3. Microbiome Composition

**Figure 1:**
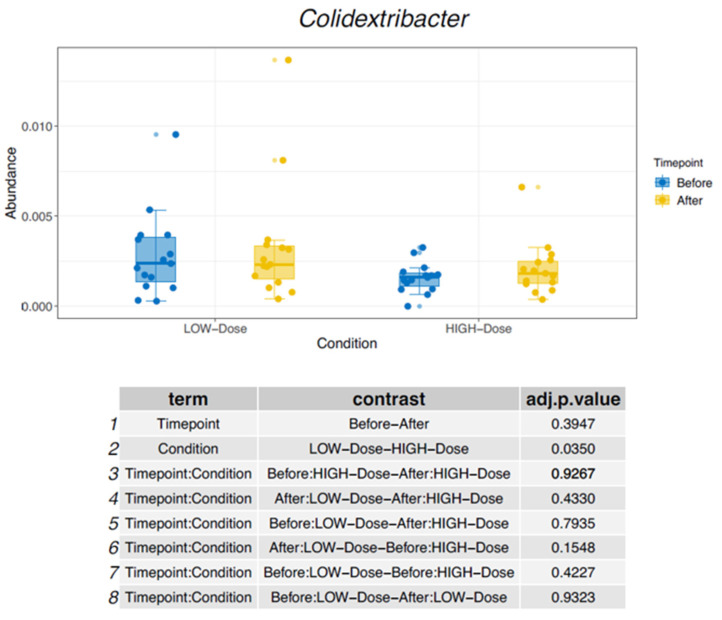
Changes in relative abundance of *Colidextribacter*.

After four weeks of oral iron supplementation, differential alterations in intestinal microbiome composition were observed. At the genus level, the relative abundance of *Colidextribacter* decreased in participants receiving low-dose iron supplementation but increased in those receiving standard-dose iron supplementation, as shown in Figure 1.

**Figure 2:**
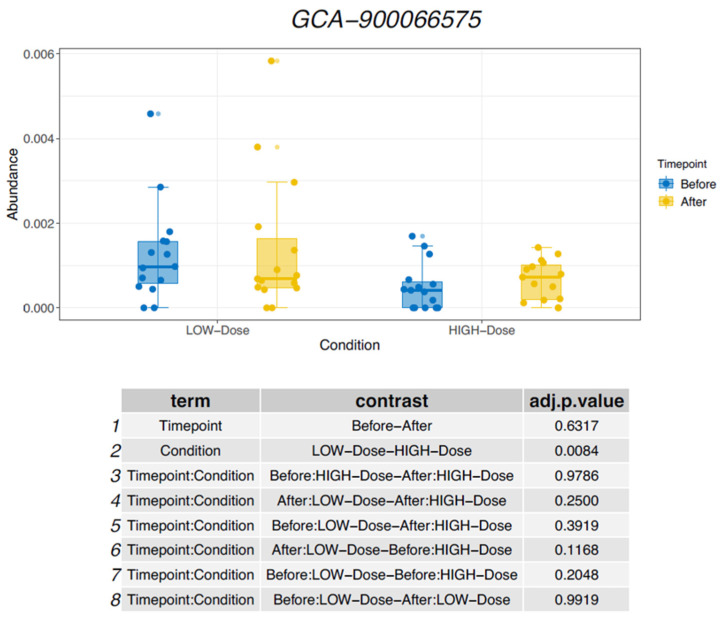
Changes in relative abundance of *GCA-900066575*.

**Figure 3:**
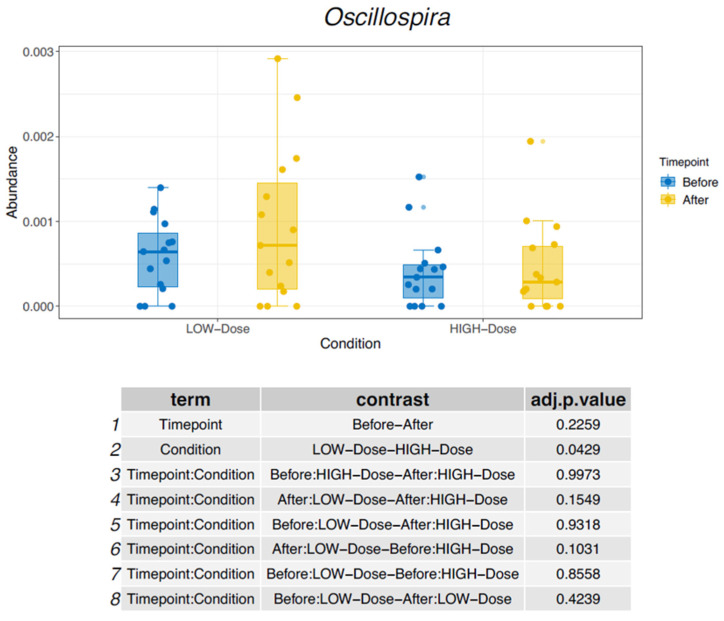
Changes in relative abundance of *Oscillospira*.

A similar pattern was observed for the genus *GCA-900066575*, which decreased in the low-dose group and increased in the standard-dose group, as shown in Figure 2. In contrast, the genus *Oscillospira* showed the opposite trend, with an increase in relative abundance under low-dose iron supplementation and a decrease under standard-dose supplementation as shown in Figure 3. Additional changes in bacterial composition across different taxonomic levels are summarized in Table 2.

### 3.4. Gastrointestinal Adverse Events

Gastrointestinal adverse events occurred substantially more frequently in participants receiving standard-dose iron supplementation. Thirteen of fifteen participants (87%) in the standard-dose group reported at least one gastrointestinal adverse event, compared with one of fifteen participants (7%) in the low-dose group (*p* < 0.0001). The most frequently reported adverse event was altered stool consistency, particularly watery stool. Other reported symptoms included diarrhea, nausea and constipation, as shown in Table 3.

## 4. Discussion

This randomized study demonstrates that oral iron supplementation exerts dose-dependent effects on the intestinal microbiome. Distinct alterations in microbial composition were observed between low-dose and standard-dose iron supplementation after only four weeks of treatment. These findings support the hypothesis that unabsorbed luminal iron can influence microbial community structure through changes in nutrient availability within the intestinal environment.

Iron availability is a critical factor regulating microbial growth and competition. Many bacterial species depend on iron acquisition systems to proliferate and increased luminal iron concentrations may selectively favor microbial taxa capable of efficient iron utilization [9,10]. Because intestinal iron absorption is limited, particularly at higher oral doses, substantial quantities of unabsorbed iron reach the colon where they may alter microbial ecology [12]. The divergent bacterial changes observed in this study therefore likely reflect shifts in microbial competition driven by differences in luminal iron availability.

Several of the taxa affected in this study have previously been associated with intestinal and systemic disease. The genus *Colidextribacter* has been linked to impaired intestinal barrier function and increased oxidative stress and has been observed more frequently in individuals with Crohn’s disease [15,16]. The genus *GCA-900066575*, first described in 2021, has been implicated in liver injury and has been proposed as a microbiome biomarker in hepatitis B virus-related hepatocellular carcinoma and post-hepatectomy liver failure [17]. In contrast, *Oscillospira* has been associated with beneficial metabolic characteristics and lower body mass index and has been suggested as a potential candidate for next-generation probiotic approaches [18,19].

Beyond these specific taxa, broader alterations in microbial communities may influence systemic metabolic and inflammatory processes [19,20,21]. Increasing evidence also suggests potential links between gut microbiome composition and neurological disorders, including Parkinson’s disease, mild cognitive impairment and Alzheimer’s disease [22,23]. Although the clinical significance of the microbiome changes observed in this study remains uncertain, the fact that these alterations were already detectable after only four weeks of treatment underscores the potential for commonly prescribed therapies such as oral iron supplementation to influence intestinal microbial ecosystems.

In addition to microbiome alterations, a pronounced difference in gastrointestinal tolerability between the two treatment regimens was observed. Gastrointestinal adverse events were reported by 87% of participants receiving standard-dose iron supplementation compared with only 7% receiving low-dose supplementation. These findings are consistent with previous reports demonstrating poor tolerability of conventional oral iron supplementation [6].

One possible explanation for this difference relates to the regulation of intestinal iron absorption. Previous studies have shown that oral iron intake induces a dose-dependent increase in plasma hepcidin levels, which subsequently reduces iron absorption [13,24]. Consequently, a greater proportion of iron remains unabsorbed and reaches the colon, increasing luminal iron exposure and thereby amplifying both gastrointestinal symptoms and microbiome perturbation. Lower iron doses may therefore result in more efficient iron uptake and reduce the amount of unabsorbed luminal iron reaching the colon, which may not improve treatment tolerability and limit microbiome disruption.

These observations are consistent with emerging therapeutic strategies aimed at reducing luminal iron burden. In addition to lower-dose regimens, alternate-day dosing and newer formulations such as amino acid chelates, liposomal or sucrosomial iron, and ferric maltol have been developed to enhance absorption and minimize gastrointestinal side effects [8].

Our findings align with the emerging concept that successful oral iron supplementation is not solely determined by elemental iron dose, but by the balance between systemic iron uptake and local intestinal exposure. In this context, lower-dose regimens may offer a clinically relevant strategy to improve tolerability while minimizing unintended effects on the gut ecosystem.

Several limitations should be considered when interpreting these findings. Although participants were instructed to maintain their usual routine, including dietary habits, these factors were not controlled and may have influenced microbiome composition. In addition, the four-week intervention period may not fully capture longer-term microbiome adaptations, as oral iron supplementation is typically prescribed for several months. The different chemical composition of the iron formulations also limits direct mechanistic interpretation. Moreover, variation in administration conditions between groups, reflecting real world intake circumstances of the respective supplementation strategies, may have influenced iron absorption and should be considered when interpreting the results. Furthermore, the open-label design may have influenced the reporting of gastrointestinal adverse events and introduces a potential risk of reporting bias. Finally, the relatively small and homogeneous study population limits the generalizability of the results.

Future studies with larger cohorts and longer follow-up periods are required to better understand the clinical implications of iron-induced microbiome alterations. In particular, studies using metagenomic or functional microbiome analyses may help clarify the biological consequences of the microbial shifts observed in response to oral iron supplementation.

## 5. Conclusions

This randomized study demonstrates that oral iron supplementation induces rapid alterations in the intestinal microbiome that differ between dosing regimens. Specific bacterial genera, including *Colidextribacter*, *GCA-900066575*, and *Oscillospira*, showed divergent changes under low-dose and standard-dose iron supplementation, indicating that luminal iron availability can influence microbial competition within the intestinal ecosystem. These microbiome changes were accompanied by pronounced differences in gastrointestinal tolerability, with substantially fewer adverse events reported in the low-dose treatment group. While the clinical significance of the observed microbiome alterations remains to be fully elucidated, the findings highlight the sensitivity of the gut microbial ecosystem to oral iron exposure. Given the widespread global use of oral iron supplementation, these results support further investigation of low-dose treatment strategies as a potentially more tolerable therapeutic approach that may also reduce microbiome perturbation.

## Figures and Tables

**Table 1:** Baseline table with *p*-values.

	Low-Dose Group (n = 15)	Standard-Dose Group (n = 15)	*p*-Value
Age (years)	23.4 ± 2.9	22.3 ± 1.7	0.22
BMI (kg/m^2^)	21.1 ± 2.0	21.0 ± 1.8	0.88
Hb (g/L)	135.5 ± 8.4	137.0 ± 7.4	0.46
Serum Ferritin (µg/L)	18.1 ± 7.4	18.4 ± 5.5	0.91
CRP ^1^ (mg/L)	1.1 ± 1.2	0.8 ± 0.7	0.36

^1^ Values below the detection limit of <0.6 mg/L were set to 0.6 mg/L for statistical analysis.

**Table 2:** Statistically significant alterations in intestinal bacterial composition during low-dose and standard-dose iron supplementation over four weeks.

Name	Taxonomy	*p*-Value	Relative Abundance Low-Dose Group	Relative Abundance Standard-Dose Group
*Erysipelatoclostridiaceae*	Family	0.036	Decrease	Increase
*Coprobacter*	Genus	0.017	Decrease	Decrease
*Bacteroides Eggerthii*	Species	0.046	Decrease	Increase
*Uncultured Alistipes*	Species	0.011	Decrease	Increase
*Alistipes Obesi*	Species	0.019	Increase	Decrease

**Table 3:** Adverse events under low-dose and standard-dose iron supplementation over four weeks.

Adverse Events	Low-Dose Iron (12 mg) (n = 15)	Standard-Dose Iron (100 mg) (n = 15)	*p*-Value ^1^
Patients reporting events, n (%)	1 (7)	13 (87)	<0.0001
Number of adverse events, n	1	15	<0.0001
Reported adverse events			
▪ Watery stool, n (%)	1 (7)	7 (47)	
▪ Diarrhea, n (%)	0	4 (27)	
▪ Nausea, n (%)	0	2 (14)	
▪ Constipation, n (%)	0	2 (14)	

^1^ *p*-values were calculated by χ^2^ test, or Mann–Whitney U-test (number of events per patient).

## Data Availability

The datasets generated and analysed during the current study are not publicly available due to ethical and data protection constraints, as they contain sensitive human microbiome data. However, anonymized sequencing data and relevant metadata supporting the findings of this study are available from the corresponding author upon reasonable request.
